# Validating the Johns Hopkins ACG Case-Mix System of the elderly in Swedish primary health care

**DOI:** 10.1186/1471-2458-6-171

**Published:** 2006-06-28

**Authors:** Anders Halling, Gerd Fridh, Ingvar Ovhed

**Affiliations:** 1 Blekinge Institute for Research & Development, Karlshamn, Sweden; 2 Department of Primary Health Care, Blekinge County Council, Karlskrona, Sweden

## Abstract

**Background:**

Individualbased measures for comorbidity are of increasing importance for planning and funding health care services. No measurement for individualbased healthcare costs exist in Sweden. The aim of this study was to validate the Johns Hopkins ACG Case-Mix System's predictive value of polypharmacy (regular use of 4 or more prescription medicines) used as a proxy for health care costs in an elderly population and to study if the prediction could be improved by adding variables from a population based study i.e. level of education, functional status indicators and health perception.

**Methods:**

The Johns Hopkins ACG Case-Mix System was applied to primary health care diagnoses of 1402 participants (60–96 years) in a cross-sectional community based study in Karlskrona, Sweden (the Swedish National study on Ageing and Care) during a period of two years before they took part in the study. The predictive value of the Johns Hopkins ACG Case-Mix System was modeled against the regular use of 4 or more prescription medicines, also using age, sex, level of education, instrumental activity of daily living- and measures of health perception as covariates.

**Results:**

In an exploratory biplot analysis the Johns Hopkins ACG Case-Mix System, was shown to explain a large part of the variance for regular use of 4 or more prescription medicines. The sensitivity of the prediction was 31.9%, whereas the specificity was 88.5%, when the Johns Hopkins ACG Case-Mix System was adjusted for age. By adding covariates to the model the sensitivity was increased to 46.3%, with a specificity of 90.1%. This increased the number of correctly classified by 5.6% and the area under the curve by 11.1%.

**Conclusion:**

The Johns Hopkins ACG Case-Mix System is an important factor in measuring comorbidity, however it does not reflect an individual's capability to function despite a disease burden, which has importance for prediction of comorbidity. In this study we have shown that information on such factors, which can be obtained from short questionnaires increases the probability to correctly predict an individual's use of resources, such as medications.

## Background

During the 20^th ^century there has been a dramatic change in demography of the western world, with an increasing elderly population. The group of elderly >80 years is now the fastest growing segment of the population [1]. With increasing age, there is an increased risk of having one or more chronic diseases [2,3]. People with multiple chronic conditions take up a large part of the healthcare resources [4]. Significant progress has been made in the development of therapies, disease management and patient education, but with rare exceptions they have been focused on single chronic conditions [5]. In Sweden where the healthcare system has centered around hospital care, there has recently been a change in policy with an increasing lean towards primary health care to provide health care for the elderly population. Today it is not possible from routine health care statistics to get an overall picture of care provided to patients with multiple chronic conditions. In the Swedish healthcare system, which has become increasingly specialised this patient group is dependent on several different caregivers who often have limited communication and consensus in planning for management of clinical care. This has resulted in a clouded picture, where the need of the patient could be unevenly distributed between different needs or fall between providers, due to lack of coordination and knowledge of the individuals overall needs. It has become increasingly important to establish a system to find and characterise patients with multiple chronic conditions as a growing body of research suggests that coordinated care (case management) results in better outcomes at lower costs [6]. The Johns Hopkins Case-Mix system [7] uses the individual as the unit of analysis for clinical and population purposes, which scores the degree of comorbidity from diagnoses during a time period, obtained from electronic patient records. Our hypothesis was that a measurement of comorbidity must take into account not just burden of chronic conditions, but also other aspects that may be of importance in characterising an individual's comorbidity such as the individual's age, sex, level of education, functional status indicators, health perception and attitudes towards future health.

Polypharmacy [8,9] is associated with treatment of multiple chronic conditions and represents the patient's doctor(s) assessment of the patients overall burden of disease. In the elderly it also represents a significant part of the cost for care. Since individual costs for care is not available in Sweden, as is the case also in many European countries because the funding is provided at the population level through national taxation, polypharmacy was chosen as a proxy measurement of care provided with which comorbidity was compared using the Johns Hopkins ACG Case-Mix system.

The aim of the study was to validate the Johns Hopkins ACG Case-Mix System in Swedish primary health care and to study if additional individual based data i.e. age, sex, level of education, activity of daily living (ADL), instrumental activity of daily living (IADL) and health perception, obtained from questionnaries in a population study, could improve the prediction.

## Methods

### Study population

The Swedish National study on Ageing and Care (SNAC) is a populationbased-, multicenter-, cohort study, which started enrollment of subjects in 2001. The study has four participating centers. One of the centers is SNAC-Blekinge which encompasses the Karlskrona community situated in south-eastern Sweden with 60600 inhabitants. This study includes baseline data collected between April 2001 and May 2003. A random, age stratified sample was selected from the population registry among those aged 60, 66, 72, 78, and 81 years. Among those aged 84, 87, 90, 93, and 96 years the entire population was included. Details of the study has been described earlier [1]. Approval was obtained from the Research Ethical Committee at Lund University, Sweden. Informed consent was obtained from all participants.

### Dependent variable

#### Drug use

Data on drug use were obtained from the participant interview. The participants were also asked to show containers, prescriptions and medication lists. If the participant was unable to give information, it was obtained from a relative, caregiver, medical staff or from prescription lists for those using the APODOS (Swedish Pharmacy Inc.) drug dispensing system. Drug use was defined as the use of drugs on a regular basis at the time of the interview and as needed at any time during the preceding month. Alternative medicines and over-the-counter drugs were also registered, but were not used in the analyses. Drugs were classified according to the Anatomical, Therapeutic and Chemical (ATC) classification system as recommended by the WHO [10]. In Sweden funding of healthcare is done at the population level. No information exist on health care cost exist on an individual level. Use of prescribed drugs represents a substantive part of the care for elderly and represents the patients doctor(s) assessment of the patients overall burden of disease. Polypharmacy was defined as the regular use of four or more prescription medicines. The dependent variable was dichotomized 1 (regular use of four or more prescription medicines) or 0 (regular use of less than four prescription medicines).

### Independent variable

#### Comorbidity

The Johns Hopkins ACG Case-Mix System is based on the theory that comorbidity, measured as a sum of diagnoses from a time period using special algorithms for grouping corresponds to a certain need for health care resources. Information about the Johns Hopkins ACG Case-Mix System, when used in Swedish primary health care and the grouping algorithms has been published earlier [11,12]. Briefly, the end result of the grouping is that each patient is allotted to one of 82 ACG groups, depending on his/her types of morbidity that are characterized using five criteria 1. Likely persistence of the condition 2. Severity of the condition 3. Aetiology 4. Diagnostic certainty and 5. Need for speciality care. Thus each ACG describes patients with the same type and degree of comorbidity. Diagnoses were obtained from electronic patient records of the three primary care districts in Karlskrona community of the participants and the ACG Case-Mix system 6.0 was applied to the diagnoses in a period of up to two years before they participated in the baseline study of SNAC-Blekinge. For analysis purposes the ACGs were collapsed into six categories (0–5) of resource utilisation bands (RUB), a higher number indicating higher morbidity. The RUB for each participant was obtained from the output of the ACG software, where 0 indicated no need for primary health care resources and 5 very high need for primary health care resources [7].

### Covariables

The research data was obtained from questionnaire's, that were part of the SNAC-Blekinge study. The scales used in this study were analysed to assess their reliability using the raw item scores. Using the 'alpha' command which analyses all items of a scale and in addition to calculating Cronbach's α [13] generates a standardised summative measure (mean 0 and standard deviation 1) of each scale into a new variable. If necessary the 'alpha' command automatically reverse the items of the scales. Thus a new composite variable was made from each scale and was used as covariates in the analysis.

### Gender

Age when the individual participated in the baseline study.

Level of education: the highest educational level of each participant was registered and categorised in the following categories; incomplete public school, 6 years of public school, 9 years of public school, complete college education, college education plus one year of post college education, complete university degree, complete PhD degree.

Activity of daily living (ADL) consisted of the following items: movement indoors, toilet, mobility from bed to chair, dressing/undressing, continence, eating [14].

Instrumental activity of daily living (IADL) consisted of the following items: cooking, laundry, use of public transportation, bathing, shopping for food, household shores, mobility outdoors [15].

Depression was measured through the Montgomery Åsberg Depression Rating Scale inventory [16]. The MADRS was specifically constructed for measuring depression and be sensitive to treatment effects. It includes 10 items and its range is between 0–60. In the present study 9 of the 10 items were used, hence yielding a range from 0–54. The MADRS has previously demonstrated to be a robust and valid measure of depression [17].

For studying self-estimated health a questionnaire (SCB) developed by Statistics Sweden for use in: Surveys of Living Conditions in Sweden (ULF) was used [18].

Attitudes towards future health was determined using part of a larger questionnaire on different aspects of health perception developed and validated by Ware et al [19].

### Statistical analysis

Independent variables to be included in a model were explored using biplots [20], where the variance explained by the variable is proportional to the length of the lines and the correlation between different independent variables is proportional to the cosine of the angle between the lines. Logistic regression was used to fit a model with polypharmacy, as the dependent variable and RUB as the independent variable. Sample weights were used to adjust for disproportionate sampling between the age groups. The model was adjusted for age (model A), age and sex (model B) age, sex, level of education and IADL (model C), age, sex, level of education, IADL, SCB and Ware (model D) in consecutive steps. Odds ratios for polypharmacy at different levels of RUB were calculated for each model. To evaluate the importance of RUB for predicting the outcome a model E consisting of model D but excluding RUB was made. The performance of model A-E for discriminating users and nonusers of polypharmacy was assessed and compared with receiver operating characteristic analysis (ROC). To assess the predictive capacity of the model, we calculated the concordance rate between predicted and observed responses and used the Hosmer-Lemeshow goodness-of-fit test [21]. All analyses were done using statistical package Stata version 9.1 (Stata Corporation, Texas, USA).

## Results

A total of 2312 individuals were invited to participate in the study. In total, 1402 (60.5%) of those invited agreed to participate. Of these 41.7% were men and 58.3% women. The participation rate was 75.2%, 66.3%, 53.7% and 47.0% for the age groups 60–69, 70–79, 80–89 and 90-years respectively. The proportion of participants in the study with different needs of primary health care and use of polypharmacy is shown in table 1. Of the participants 1381 had given information on their use of prescription drugs and a further 237 had missing values on the independent variables, table 2. This left 1144 participants with complete datasets, who participated in this study.

The biplot analysis showed that ADL was highly correlated with IADL and MADRS to age. Due to risk of problems with multicollinearity these variables were omitted from the models. The odds ratio for having polypharmacy was significantly increased in RUB 3 and 4, when compared with RUB 0, table 3. The number of participants in RUB 5 were small and the confidence intervall large. However in model A-C, those in RUB 5 had a higher need for polypharmacy. In further analysis the ability of the Johns Hopkins ACG Case-Mix system to discriminate between users and nonusers of polypharmacy and if it could be improved was tested by adding independent variables to the model in subsequent steps, table 4. The univariate model is not presented as missingness of data was age dependent and could violate missing data assumptions. By adding independent variables to the model the sensitivity rose by 14.4%, with a 1.6% decline in specificity. The number of correctly classified rose by 5.6% and the area under the curve by 11.1%. Using ROC analysis model D was shown to have a significantly better discriminatory power when tested against models A-C (p < 0.001) or model E (p < 0.001), where RUB had been excluded. The predictive models that included RUB all had a good fit, whereas model E provided a poor fit.

## Discussion

In this study we have shown that the Johns Hopkins ACG Case-Mix system is a major factor in the model explaining the proxy variable polypharmacy. We have also shown that the sensitivity of the Johns Hopkins ACG Case-Mix, when used with Swedish primary health care diagnoses could be improved. This finding is not surprising since primary health care diagnoses provide little information on the severity of the diagnosis, the individuals ability to function despite a disease burden, the patient's health perception or his/her attitudes towards future health. By adding information on the biologic severity of the diagnoses in subsequent steps, the individual's age, sex, level of education, functional status indicators and healthperception, we were both able to improve the sensitivity and discriminatory power of the prediciton of the proxy variable polypharmacy. Only models that included RUB provided a good fit. We especially found that information on an individual's IADL function and self estimated health complement the information given by the Johns Hopkins ACG Case-Mix together with age and gender on the level of the individual's comorbidity.

The data for this study was derived from a population based study and a database of diagnoses from the electronic patient record system in primary health care. The participation rate in the population study were lower in the older age groups and many of these non participants voluntarily reported that they were either to old or sick to participate, which is likely to have led to an underestimation of comorbidity in the oldest age group. Also missing values in independent variables of the self-administered questionnaires increased with age. The diagnoses used in this study was obtained from registers used in routine medical care, without any additional training given to the primary health care physicians. It is important to know that the Johns Hopkins ACG Case-Mix system is sensitive towards number of diagnoses used per visit and that diagnostic labelling by different physicians has been shown to vary [22]. In addition there are two private primary care physicians in the area, whose diagnostic registers were unavailable for us, which thus would have lowered the total comorbidity to a small degree. The diagnoses that we used for calculations by the Johns Hopkins ACG Case-Mix system were collected up to two years prior to their day of participation, which has been suggested to be optimal for the Swedish primary health care system where all chronic conditions are not registered each year [23].

A previous Swedish study has shown that the Johns Hopkins ACG Case-Mix explain more of the variation in health care cost than age and gender alone [24]. This study has shown that the prediction of comorbidity can be improved by adding information on functional status indicators which is in agreement with Mayo et al. [25] and that it can be further improved by adding selfestimated health. The variance in health care cost explained by the Johns Hopkins ACG Case-Mix system in a Canadian and Spanish study was larger than in a Swedish study, which might be due to differences in reimbursement system and diagnostic practice [26,27].

There is a need for an individualbased measure of comorbidity both for funding of medical care and for planning of preventive- and medical services. Such a measure should have as high validity as possible and be easy to use in practical care. The Johns Hopkins ACG Case-Mix system provides such a system, which has been extensively validated both in the USA, Canada and in Europe [28-30]. Our study show that other measures complement the information derived from this diagnosis based system in the prediction of comorbidity. In practical care it may also be of importance to have a comorbidity measure, which is derived from different sources so as to minimise the effect of different diagnostic practices by different doctors and so called 'diagnostic gliding'.

Comorbidity provides new challenges to health care services that have traditionally been focused on individual diseases and with little substantial collaboration between primary care physicians and other specialist physicians.

During the last century demography has changed radically with moderate changes in the health care system. One of the challenges resulting from the successes of both preventive measures, better food and curative health services is the increase in the extent of comorbidity. Many elderly patients now receive health care services from many different caregivers with little substantial collaboration. Some forms of patient-oriented case management are specifically designed to enhance the coordination of care on the grounds that the common characteristics of successful care for major chronic diseases as well as for preventive activities provide strong arguments for care to be coordinated by primary care phycicians [31]. These primary care physicians however need to have the tools that easily identify patients with comorbidity. Use of the Johns Hopkins ACG Case-Mix system together with a geographical information system provides an interesting idea to visualise comorbidity [32].

Traditionally treatment programs have been focused on individual diseases, whereas in reality 50% of all individuals with chronic conditions have multiple chronic conditions [33]. Individuals with multiple chronic conditions have clinical needs that may differentiate them from persons with a single chronic condition. Evidence indicates that chronic conditions cluster and that persons with multiple chronic conditions may have more rapid declines in health status and a greater likelihood of disability. Use of a valid measure of comorbidity and especially if it also could be a measure of different aspects of comorbidity that could be demonstrated on different axis in a star diagram could be of value in guiding the out patient clinician and discharging doctor to appropriate treatment programs. Relatively recently a treatment program not directed to a specific disease has been shown to improve health status and reduce hospitalisation [34].

The results of this study would have been improved with diagnostic training of the primary care physicians in the study, although we believe it unlikely that the variation due to this can be totally eliminated, which is why we suggest this comorbidity measure based on additional different factors. Work on establishing Patient-level Clinical [35] costing in primary health care is currently ongoing in Blekinge county and further work will be directed at studying if this measure holds in other clinical settings.

## Conclusion

This study was done to validate the Johns Hopkins Case-mix system in Swedish primary health care and to study if additional information from short questionnaires could increase the information of the individual's comorbidity compared to that estimated by the Johns Hopkins Case-mix system alone. In this study we have shown that information on these factors, increases the probability to correctly predict an individual's comorbidity. This increased the number of correctly classified by 5.6% and the area under the curve by 11.1%. Therefore we suggest that measures of comorbidity, which with relative ease could be obtained from a population using questionnaires, must include different dimensions of health and functioning.

## Competing interests

The author(s) declare that they have no competing interests.

## Authors' contributions

AH conceived the idea and designed the study, collected all the data, handled the ACG software, performed the statistical analyses and wrote the manuscript. GF helped to co-ordinate the work and drafted the manuscript. IO participated in the design of the study, drafted the manuscript and helped to co-ordinate the work.

All authors read and approved the final manuscript.

## Pre-publication history

The pre-publication history for this paper can be accessed here:



## Figures and Tables

**Table 1:** Proportion of population with a specific degree of utilisation of primary health care and polypharmacy by age and gender.

**Age (years)**	RUB^a^	60–69 n = 206 %	70–79 n = 192 %	80–89 n = 318 %	90–96 n = 88 %
**Female**	0	59.7	21.9	19.2	36.4
	1	3.4	4.2	8.2	11.4
	2	15.0	21.3	19.8	17.0
	3	18.4	46.9	45.3	28.4
	4	3.4	5.7	6.3	6.8
	5	0	0	1.3	0
					
Polypharmacy (%)^b^		19.4	34.4	50.3	58.0

**Age (years)**	RUB	60–69 n = 186 %	70–79 n = 148 %	80–89 n = 207 %	90–96 n = 36 %

**Male**	0	64.5	21.6	23.7	22.2
	1	2.1	6.8	7.3	5.6
	2	14.5	25.7	23.7	16.7
	3	17.2	38.5	39.6	47.2
	4	1.6	6.1	4.8	8.3
	5	0	1.4	1.0	0
					
Polypharmacy (%)		14.5	29.7	39.1	61.1

^a ^Resource utilisation band (RUB) according to the Johns Hopkins Case-mix system.

^b ^Polypharmacy: regular use of four or more prescribed medications.

**Table 2:** Prevalence of missing values in independent variables.

**Age (years)**	60–69 %	70–79 %	80–89 %	90–96 %
**Female**				
Level of education	1.9	4.2	13.1	21.1
IADL function	0.5	0	1.0	0
SCB	2.4	4.7	16.5	37.6
Ware	2.4	6.3	20.2	43.1
				
**Male**				
Level of education	3.3	5.4	11.0	21.4
IADL function	0	0.7	0.5	0
SCB	3.2	6.1	13.0	38.1
Ware	4.8	8.1	15.0	40.5

**Table 3:** Odds ratios (OR) and 95% confidence intervals (CI) for use of polypharmacy (4 or more regular prescription medicines) according to resource utilisation band (RUB).

RUB	OR	model A 95%CI n = 1144	OR	model B 95%CI n = 1144	OR	model C 95%CI n = 1144	OR	model D 95%CI n = 1144
0	1.0		1.0		1.0		1.0	
1	0.9	0.4–1.8	0.9	0.4–1.7	0.9	0.5–1.9	1.0	0.5–2.1
2	1.2	0.8–1.9	1.2	0.8–1.9	1.3	0.8–2.1	1.3	0.8–2.1
3	3.0***	2.1–4.4	3.0***	2.0–4.4	3.3***	2.2–4.9	3.0***	2.0–4.6
4	6.8***	3.5–13.5	6.7***	3.4–13.2	8.2***	4.0–16.8	6.5***	3.1–13.6
5	6.4*	1.2–35.2	7.0*	1.2–39.6	7.8*	1.5–41.0	4.8	1.1–21.3

* significant at 5%, ** significant at 1%, *** significant at 0.1%

model A: adjusted for age

model B: adjusted for age and sex

model C: adjusted for age, sex, level of education and IADL function

model D: adjusted for age, sex, level of education, IADL function, SCB and Ware

**Table 4:** Sensitivity, specificity, positive- and negative predicitive value (%) for detecting use of polypharmacy (4 or more regular prescription medicines) according to resource utilisation band (RUB).

	model A	model B	model C	model D	model E
Sensitivity	31.9	33.6	38.4	46.3	41.2
Specificity	88.5	89.2	89.8	90.1	90.2
Positive predicitive value	55.4	58.3	62.7	67.8	65.5
Negative predicitive value	74.4	75.0	76.5	78.9	77.4
Correctly classified	71.0	72.0	73.9	76.6	75.1
Area under ROC curve	0.72 (0.69–0.75)^a^	0.73 (0.70–0.76)	0.76 (0.73–0.79)	0.80 (0.77–0.82)	0.77 (0.74–0.80)
Goodness-of-fit	0.90	0.39	0.64	0.31	0.01

^a ^95% confidence interval

model A: adjusted for age

model B: adjusted for sex and age

model C: adjusted for sex, age, level of education and IADL function

model D: adjusted for sex, age, level of education, IADL function, SCB and Ware

model E: model D excluding RUB
